# How comprehensive is our comprehensive geriatric assessment in clinical practice? An Irish perspective

**DOI:** 10.1007/s41999-024-00973-4

**Published:** 2024-04-22

**Authors:** Karen Dennehy, Amy Lynch, Catriona Reddin, Bart Daly, Tim Dukelow, Michelle Canavan, Maria Costello, Robert Murphy

**Affiliations:** 1 Cork University Hospital, Cork, Ireland; 2 Galway University Hospital, Galway, Ireland; 3 University of Galway, Galway University Hospital, Galway, Ireland; 4 University College Cork, Cork, Ireland

**Keywords:** Integrated care, Comprehensive geriatric assessment, Population health policy, Minimum dataset, Standardisation of practice

## Abstract

**Purpose:**

Comprehensive geriatric assessment (CGA) is the cornerstone of high-quality care for older adults. There is no current gold standard to guide what should be included as the baseline measure for CGAs. We examined what metrics are being captured in CGA baseline assessments completed by community based integrated care teams in Ireland.

**Methods:**

CGA’s care pathways in Ireland are usually initiated with a written document that establish patients baseline in various assessment areas. These documents were the focus of this study. We completed a cross-sectional study of the components captured in CGA baseline assessments completed in a community setting. We contacted operational leads in each of the community health organisations in Ireland and requested a copy of their current initial baseline screening document for CGA.

**Results:**

We reviewed 16 individual CGA baseline documents for analysis in this study. Common assessment areas in all documents included frailty (with the Rockwood Clinical frailty scale used in 94%, *n* = 15), cognition (4AT—56% of CGAs, MMSE—25%, MOCA—25%, AMTS—19%, AD8—19%, Addenbrookes—13%, 6CIT—13%, mini cog—6%), mobility (100%, *n* = 16), falls (100%, *n* = 16), continence (100% *n* = 16), nutrition (100% *n* = 16). Mood (94%, *n* = 15), pain (44%, *n* = 7), bone health (63%, *n* = 10), sleep (62%, *n* = 10) and skin integrity (56%, *n* = 9). Formal functional assessment was completed in 94% (*n* = 15) of CGAs with the Barthel index being the tool most used 81% (*n* = 13). Half of the CGAs included a section describing carer strain (50%, *n* = 8). The majority of CGAs included a patient centred question which was some variation of ‘what matters most to me’ (75% *n* = 11). 87.5% of assessments included a care plan summary (*n* = 14).

**Conclusions:**

This report highlights that the core tenets of CGA are being assessed across different community based initial CGA screening instruments. There was significant variability in the discussion of challenging topics such as carer strain and social well-being. Our results should prompt a discussion about whether a minimum dataset should be developed for inclusion in nationwide initial baseline CGA document, aiming to improve standardisation of assessments, which will impact areas highlighted for intervention and ultimately guide population health policy.

**Supplementary Information:**

The online version contains supplementary material available at 10.1007/s41999-024-00973-4.

## Key summary points

**Aim:**

To exam the metrics being captured in CGA baseline assessment throughout Ireland in integrated care teams.

**Findings:**

16 individual CGA baseline documents were analysed, core tenets are being captured, however disparity exists with regards challenging topics and patient centered care.

**Message:**

A nationwide CGA document could improve standardization of assessments and guide population health policy.

**Supplementary Information:**

The online version contains supplementary material available at 10.1007/s41999-024-00973-4.

## Background

Comprehensive geriatric assessment (CGA) tools evaluate older individuals, with the aim of identifying health issues, formulating coordinated management plans, and improving health outcomes [1]. CGA enables healthcare professionals to effectively address the needs and goals of older adults, with the ultimate objective of supporting independent living at home [2]. In community settings, there is a growing body of evidence that shows completing a CGA with complex interventions reduces hospital admissions, readmissions, and frailty [3, 4].

We sought to examine the specific content captured during the baseline assessments which form part of the CGA process in newly developed integrated care teams across the Republic of Ireland. Integrated care underpinned by a health and well-being approach, is central to the Ireland’s health service reform [5]. This new community healthcare network is in the process of implementation across Ireland as part of the enhanced community care (ECC) Programme, this is to change the healthcare model of Ireland towards community-based interventions by enhancing resources closer to people’s homes, thereby reducing the pressures on hospital services [5]. Older people are a particular focus of the ECC programme and the integrated care programme for older people (ICPOP) community specialists teams are centrally positioned to provide quality care to older persons living in the community [6, 7].

All teams work within a community health organisation (CHO) in Ireland. There are 9 CHO in Ireland, which have been divided geographically, including both urban and suburban areas. There may be more than one integrated care teams working within a CHO and there are multiple CGA documents in use. Integrated care teams work in the community but receive referrals from several sources, including general practice, emergency departments, other specialities or upon discharge from an acute hospital admission. Integrated care teams as outlined below are composed of several interdisciplinary specialists and manage common syndromes, including falls, frailty, movement disorders, cognitive decline, mobility issues and bone health.

These community teams’ (ICPOP) delivery of CGA often begins with a paper-based document which is used as a baseline assessment, these documents are the focus of this cross-sectional study. CGA’s care pathways in Ireland are usually initiated with a written document that establish patients baseline in various assessment areas, this helps to identify any issues before a personalised management plan is enacted and health outcomes improved. These documents are the initial assessment and are often completed by various health and social care professionals, including medical, nursing, and allied health professionals (including occupational therapists, physiotherapists, speech and language therapists, dieticians, social workers, psychologists).

These paper-based documents are the focus of this study, they captures baseline information on initial contact with an older person is often updated as necessary throughout later care episodes. It has been highlighted that the term CGA is a misnomer. Beyond the assessment, the integrated care plan hence created is an integral part of the process, the information captured using these paper based documents in the initial meeting only forms part of the CGA process [8].

Team members can vary between ICPOP hubs. In this study we did not record the exact numbers of each discipline in every community team, but in general they are made up of the afore mentioned interdisciplinary members, nursing and medical personnel.

Ireland’s population is ageing faster than anywhere else in Europe and there are more people in Ireland living longer lives than before [9]. We need to be proactively identifying health status and predicting issues that may arise rather than traditional access points such a general practitioner or emergency departments. This shift in healthcare delivery is to try and tackle these challenges and effective use of CGAs can be used to assist this.

However, despite the clear benefit of CGA there is a lack of standardised practice in the day-to-day delivery of CGAs and no gold standard exists [10]. Defining the core elements that exist in current CGA documents and examining the different assessment tools in use has implications for standardisation of practice.

## Methods

We completed a cross sectional study examining what domains were included in different CGA baseline assessment documents completed by community based integrated care teams. Each integrated care team operates within one of nine separate community health organisations. We contacted operational leads from all nine organisations to request a copy of their current paper based CGA document.

We examined what components were included across the core domains of well-being. This included physical health (co-morbidities, medications), psychological health (including both cognitive and affective status), functional (basic and instrumental activities of daily living and mobility) and socioenvironmental well-being (including social networks, safety, environmental safety) [11]. These core domains are well-established since the inception and widespread use of CGA over that past number of decades [12]. We developed an extraction key. All data were extracted separately by two independent reviewers.

We documented all aspects of physical, psychological, functional, and socioenvironmental well-being that were recorded in the CGA documents. We documented if a care plan summary or action list was included.

## Results

We received responses from eight of the nine community health organisations, resulting in 16 different CGA paper-based baseline documents available for review. All CGA care pathways start with a paper-based document used to capture the relevant information to form part of their assessments. The median length of the paper-based documents in use was 14 pages with a range from 4 to 28 pages. Common areas in all CGAs included assessments of physical, psychological, functional and socio environmental well-being.

### Physical assessment

The majority of CGAs (81%, *n* = 13) included a section for recording the past medical history—some using a tick box (23%, *n* = 3) with the remaining using free-text entries (77%, *n* = 10). Medication review was included in 94% (*n* = 15). Continence and nutrition were addressed in every CGA.

The formal assessment tools used to assess nutritional status included—the malnutrition universal screening tool (44%, *n* = 7), the mini nutritional assessment short form (25%, *n* = 4) and the malnutrition screening tool (19%, *n* = 3). Pain assessment was included in 44% (*n* = 7) of all CGA documents, the tool used to assess pain included the visual analogue scale (31%, *n* = 5) and the numeral rating scale (6%, n = 1). A section on skin integrity was included in just over half of all CGAs (9/16), of these 5 documents used a formal instrument—the waterlow score (31%).

Bone health was addressed in 63% (*n* = 10) of all CGA’s, of these 5 used the formal FRAX tool (31%). The Rockwood Clinical frailty scale was applied to all documents that assessed frailty 94% (n = 15 CGAs) (Table 1). There was a prompt for sarcopenia in 12 documents (75%), 10 documents captured grip strength (63%, n = 10), while two documents used the SARC-F tool (13%).

**Table 1:** Physical Assessment

	CGA 1	CGA 2	CGA 3	CGA 4	CGA 5	CGA 6	CGA 7	CGA 8	CGA 9	CGA 10	CGA 11	CGA 12	CGA 13	CGA 14	CGA 15	CGA 16
Frailty	**X**	**X**	**X**	**X**	**X**	**X**	**X**	**X**	**X**	**X**	**X**	**X**	**X**	**X**	**X**	
Rockwood CFS	**X**	**X**	**X**	**X**	**X**	**X**	**X**	**X**	**X**	**X**	**X**	**X**	**X**	**X**	**X**	
Sarcopenia	**X**	**X**	**X**	**X**	**X**	**X**		**X**	**X**	**X**	**X**	**X**				**X**
SARC-F					**X**											**X**
Grip Strength	**X**	**X**	**X**	**X**		**X**		**X**	**X**	**X**	**X**	**X**				
Bone health	**X**	**X**	**X**	**X**				**X**			**X**	**X**	**X**		**X**	**X**
FRAX	**X**		**X**								**X**		**X**		**X**	
Pain		**X**	**X**	**X**	**X**	**X**				**X**	**X**	**X**				
NRS											**X**					
VAS			**X**		**X**	**X**				**X**		**X**				
Skin integrity	**X**		**X**	**X**	**X**	**X**				**X**	**X**	**X**	**X**			
Waterlow Score			**X**		**X**	**X**				**X**		**X**				
Nutrition	**X**	**X**	**X**	**X**	**X**	**X**	**X**	**X**	**X**	**X**	**X**	**X**	**X**	**X**	**X**	**X**
MST	**X**						**X**							**X**		
MUST			**X**		**X**				**X**		**X**	**X**	**X**	**X**		
MNA-SF						**X**		**X**		**X**						**X**
M-SASQ					**X**											
Swallow Ax	**X**	**X**	**X**	**X**		**X**	**X**	**X**	**X**	**X**	**X**	**X**	**X**		**X**	**X**
Oral Health	**X**				**X**			**X**								**X**
Medications	**X**	**X**	**X**	**X**	**X**		**X**	**X**	**X**	**X**	**X**	**X**	**X**	**X**	**X**	**X**
Continence	**X**	**X**	**X**	**X**	**X**	**X**	**X**	**X**	**X**	**X**	**X**	**X**	**X**	**X**	**X**	**X**

*Rockwood CFS* rockwood clinical frailty scale, *sarc-F* strength, assistance in walking, rise from a chair, climb stairs and falls, *FRAX* fracture risk assessment tool, *NRS* numerical rating scale, *VAS* visual analogue scale, *MST* malnutrition screening tool, *MUST* malnutrition universal screening tool, *MNA–SF *mini nutritional assessment short form, *M-SASQ* modified single alcohol screening questionnaires (M-SASQ)

There was a swallow assessment prompt in 14 documents (88%) and oral health assessment in 4 documents (25%).

### Psychological assessment

Cognition was addressed in every document (Table 2). The tools that were prompted in the documents included; the 4AT (56%, *n* = 9), MMSE (25%, *n* = 4), MOCA (25%, *n* = 4), abbreviated mental test score (19%, *n* = 3), AD8 (19%, *n* = 3), Addenbrookes cognitive examination (13%, *n* = 2), 6CIT (13%, *n* = 2) and the mini-cog (6%, *n* = 1).

**Table 2:** Psychological Assessment

	CGA 1	CGA 2	CGA 3	CGA 4	CGA 5	CGA 6	CGA 7	CGA 8	CGA 9	CGA 10	CGA 11	CGA 12	CGA 13	CGA 14	CGA 15	CGA 16
Cognitive Ax	**X**	**X**	**X**	**X**	**X**	**X**	**X**	**X**	**X**	**X**	**X**	**X**	**X**	**X**	**X**	**X**
AMTS	**X**	**X**													**X**	
4AT		**X**		**X**		**X**		**X**		**X**	**X**		**X**	**X**		**X**
Addenbrookes	**X**											**X**				
MMSE	**X**				**X**				**X**	**X**						
MOCA	**X**	**X**		**X**					**X**							
AD8					**X**					**X**			**X**			
Mini-Cog							**X**									
6 CIT score									**X**							**X**
Mood	**X**	**X**	**X**	**X**	**X**	**X**	**X**	**X**	**X**	**X**	**X**	**X**	**X**		**X**	**X**
GAD 7 Scale	**X**															
Geriatric 4-item dep. scale			**X**													
GDS	**X**	**X**	**X**	**X**	**X**	**X**			**X**		**X**	**X**	**X**			**X**
Cornell scale					**X**	**X**										
Sleep	**X**	**X**	**X**	**X**	**X**	**X**			**X**	**X**	**X**		**X**			
Adapted SDS CL 17	**X**															
Stop bang	**X**															
EQ5LD		**X**	**X**	**X**			**X**						**X**			**X**

*AMTS* abbreviated mental test score, *4AT* alertness, 4AMT, attention, acute change, *Addenbrookes* Addenbrookes cognitive assessment tool, *MMSE* mini-mental state examination, *MOCA *montreal cognitive assessment, *AD8 *8-item informant interview to differentiate aging and dementia, *6CIT screen* 6-item cognitive impairment test, *GAD 7* generalized anxiety disorder 7-item scale, *GDS* geriatric depression scale, *Adapted SDS CL 17* sleep disorder symptom checklist,* EQ-5D-5L* EuroQol 5 dimension 5 level instrument

Mood assessments were specific sections in 94% of CGAs (*n* = 15). The formal tool assessments for mood included geriatric depression scale (69%, *n* = 11), Cornell scale (13%, *n* = 2), GAD7 (6%, *n* = 1) and the geriatric 4-item depression scale (6%, *n* = 1).

Ten documents (62%) discussed sleep, of these 2 used formal sleep assessment tools which were stop bang (6%, *n* = 1) and the adapted SDS CL 17 (6%, *n* = 1).

A formal quality of life assessment tool was used in 6 CGA documents—using the EQ5LD (38%) score.

### Functional assessment

A formal functional assessment was completed in 94% (*n* = 15) of CGAs with the Barthel index being the tool most frequently used 88% (*n* = 14). Four CGAs applied the Lawton Instrumental Activities of Daily Living (IADL) Scale (25%) and one document used the Nottingham extended ADL scale (6%).

Every CGA addressed mobility, of these fives used the berg balance as a formal tool to assess patients’ mobility. All CGA documents included some prompt to discuss falls. The TUG tool was used (56%, *n* = 9), as well as FES scale (19%, *n* = 3), 5STS was utilised in three documents (19%), both the FRASE tool and FROP-Com were used once (6%, *n* = 1).

Access to modes of transport and driving status was included in 15 CGAs (94%). The ability to manage finances was captured through the Lawton IADL scale in four CGAs (Table 3).

**Table 3:** Functional Assessment

	CGA 1	CGA 2	CGA 3	CGA 4	CGA 5	CGA 6	CGA 7	CGA 8	CGA 9	CGA 10	CGA 11	CGA 12	CGA 13	CGA 14	CGA 15	CGA 16
Formal functional assessment	**X**	**X**	**X**	**X**	**X**	**X**	**X**	**X**	**X**	**X**	**X**	**X**	**X**	**X**	**X**	
Barthel index	**X**	**X**	**X**	**X**	**X**		**X**	**X**	**X**	**X**	**X**	**X**	**X**	**X**	**X**	
Lawton’s IADL	**X**				**X**	**X**	**X**									
Nottingham ext. ADL scale											**X**					
Mobility	**X**	**X**	**X**	**X**	**X**	**X**	**X**	**X**	**X**	**X**	**X**	**X**	**X**	**X**	**X**	**X**
Berg balance	**X**	**X**		**X**				**X**			**X**					
Falls	**X**	**X**	**X**	**X**	**X**	**X**	**X**	**X**	**X**	**X**	**X**	**X**	**X**	**X**	**X**	**X**
FRASE			**X**													
FROP-Com														**X**		
FES		**X**		**X**							**X**					
TUG	**X**	**X**	**X**	**X**	**X**					**X**	**X**	**X**	**X**			
5STS						**X**				**X**	**X**					

*Lawton’s IADL* Lawton’s instrumental activities of daily living, Nottingham ext. *ADL scale* nottingham extended activities of daily living scale, *FRASE* falls risk assessment scale for elderly, *FROP-Com* adapted falls risk for older people in the community screen, *FES *falls efficacy scale, *TUG* timed up and go test, *5STS* five times sit to stand

### Socio-environmental assessment

Seven of the documents (43%) specifically captured whether the patient has an enduring power of attorney in place, 5 (31%) collected information about formal advanced care directives, and 2 (12%) enquired about nominated assisted decision makers. Half of CGAs included a section describing carer strain, four of which used the formal caregiver strain index (25%). Sexual health was not explicitly addressed in any CGA.

### Purpose of assessment

The majority of CGA documents included a patient centred question which was some variation of ‘what matters most to me’ (75% *n* = 11). 87.5% of assessments included a section with a care plan summary where action areas can be highlight (*n* = 14). There were no standardised intervention protocols on the documents assessed in this study, the ‘actioning section’ of the care plan summary was always individualised based on the issues identified.

## Discussion

In this study, we identified that core assessment areas in all CGA proformas used in integrated care teams with the greatest variability in the assessment of social well-being. These core domains are well-established since the inception and wide spread use of CGA over that past number of decades [12]. The majority of CGAs reviewed comprised a comprehensive assessment of physical, psychological and functional health all of which are integral to the well-being of older persons to optimise quality of life [13]. However, the only topics that were unanimously assessed in every CGA document were—mobility, falls, continence and nutrition, this highlights that there is disparity in the patient areas assessed across our new integrated care teams.

There was significant heterogeneity in the structure of the social economic care section of CGAs across different integrated care hubs, particularly with respect to capturing information around pertinent legal issues such as advanced care planning and assisted decision making. More than half of CGAs studied failed to address these topics, thereby missing a unique opportunity to start the discussion with patients. This is especially relevant in Ireland as the Assisted Decision-Making (Capacity) Act has been enacted in 2023. The purpose of this act is to promote the autonomy of persons concerning their treatment choices to enable them to be treated according to their will and preference and to provide health professionals with important information about persons and their choices in relation to treatment [14, 15].

We noted that personal ADLs (*n* = 15) were captured much more frequently than instrumental ADLs (*n* = 2). There was significant variability in the discussion of challenging topics in such as carer strain. Discussing carer well-being is essential as this support network is key to older people remaining well in their own homes [16, 17]. The inclusion of a screening question for carer strain could serve as a trigger for more in-depth assessment or onward referral.

Sexual health was not addressed in any of the CGAs reviewed in this study. Previous systematic review have highlighted that sexuality remain important for older many people but the sense of disinterest by healthcare professional inhibits discussions around same [18]. Taking a sexual history can be challenging, however, older adults do perceive sexuality to be an important quality of life issue [19].

There were two CGA paper-based documents that did not have any documentation of areas to action or care plan summary. It is unclear how these documents achieve the goals of implementing change in pertinent areas to ensure that health outcomes are targeted. It has been highlighted that the term CGA is a misnomer. Beyond the assessment, the integrated care plan hence created and enacted is an integral part of the process [8, 20].

A review article looking at literature over a three decade period identified that the core domains of CGA are functional status, mobility, gait speed, cognition, mood and emotional status, nutritional status, comorbidities and polypharmacy, geriatric syndromes (fall risk, delirium, urinary incontinence, dentition, visual, or hearing impairments), disease-specific rating scales (i.e., parkinsonism, dementia), goals of care, and advanced care planning [20]. Social, environmental and finances are also key components. CGA uses validated geriatric scales and tests to produce an inventory of health problems, for the purposes of developing an individualized geriatric intervention plan [20]. These key components were captured in the CGA documents reviewed in our cross sectional study.

CGA’s are being adapted more frequently as a valuable tool to target a broader range of patients in other subspecialities [21]. An inpatient CGA service for the proactive care of older people undergoing surgery has been established at Guys and St Thomas’ Hospital since 2003, providing multidisciplinary team preoperative optimisation and post operative management of elective and emergency admission. This has reduced post operative complications and length of stays [22]. Another example includes CGA based targeted cardiac rehabilitation which was applied to older patients undergoing transaortic valve implantation procedure, this improved functional status pre-procedurally, including frailty, BMI and malnutrition [23]. The multidimensional prognostic index (MPI) has been developed to translate CGA findings into a numerical score predictive of one year mortality. This can be used as an objective marker to optimise management of vulnerable patients, but clinicians themselves need to take the initiative to perform and implement targeted intervention to utilise this information and improve outcomes [24, 25].

CGA is capable of effectively exploring multiple domains in older age as well as facilitating prognosis and clinical decision making for personalised care plans for older persons to improve health outcomes. There is mixed evidence around the application of CGA in the outpatient setting, however, targeting particular populations e.g., treating patients at higher risk of hospitalization has shown benefits [26].

A limitation of this research is the heterogeneity that exists in Irelands newly developed community teams and in the multi-disciplinary members contained therein. This may account for the diversity seen in the social well-being section as some teams do not have a social worker. Another limitation for this study was that we did not receive responses from all CHOs (8/9) which means that we cannot describe this study as fully representative of all the CGA documents in use currently throughout Irelands new integrated care teams. It was not feasible in this study to speak directly to CHO operational leads or directly to the clinical staff completing these CGA documents which would have added a unique and valuable perspective. A further limitation is the external validity given that CGA documents examined in this study were in the community setting and these findings are less generalisable to the broader context—for example, hospital or emergency department-based documents.

The ICOPE framework introduced by the world health organisation has provided evidence-based guidelines to healthcare providers on the approach to identify and manage decline in physical, functional, or psychological well-being of older people in the community [27]. The important steps in these guidelines are being applied to the care pathways in Ireland which include screening, patient centred assessment, personalised care plans, referral pathway/follow up and engagement with communities and caregivers. We should continue to uphold these cornerstones of optimal care by synchronizing service delivery.

The emergence of new integrated care teams in Ireland has focused on the development of new pathways to target the complex needs of our aging population. Our results highlight significant heterogeneity between CGAs, and the data collected therein. This should serve as a prompt for discussion as to whether a minimum dataset should be developed for inclusion within a nationwide CGA to improve standardisation of assessments ultimately guide policy at a population health level*.* A minimum common data set was developed for hip fractures audits across countries who have registries to develop an accepted standard for monitoring and improving services [28]. This could be applicable to geriatric medicine and CGA pathway use. However, there are challenges in achieving a consensus, in a recent study assessing geriatricians opinions on core components of CGA there was limited agreement [29].

Barriers in an Irish context to a uniform data set include the lack of technological infrastructure in integrated care team services. In Ireland there is not universal access to GP records, medication lists or electronic patient records which is also applicable to many other health systems. InterRAI, an internationally utilised assessment tool, designed to be patient-centred and provide equitable and transparent access to high quality services based on assessed care needs [30], has been piloted in some community health networks in Ireland. InterRAI is a collaborative approach by researchers in over 30 countries who have developed a tool to improve care for medically complex individuals evaluating and implementing instruments for comprehensive assessment [31]. InterRAI instruments are administered by trained clinician–assessors who interact with the person, informal caregivers and care providers. InterRAI represents an integrated health information system providing patient centred information enhancing the delivery of care as a system which may form part of the solution going forward [31]. While there are benefits in utilising this tool—standardisation, ease of use and optimisation of resources, the tool requires trained individuals to complete the assessment [30].

An umbrella review from the UK looking at hospitalised inpatients found consistencies across included studies (24 articles included) about the data, intervention, settings, and outcomes captured in CGA instruments in use [10]. However, similarly to our findings, there was a lack of patient centred care given that patient reported outcome measures were reported in only a few studies.

This study highlights that the core tenants of CGA are being assessed across different community based CGA documents. There are inconsistencies in the methods by which data are captured in CGAs which may have implications for planning onward interventions and uniformity across health services. CGA is central to the assessment of complex older adults and allows a standardised assessment, helps to identify multidisciplinary team members who may need to become involved in the older persons care and it identifies what is important to the person and what targeted intervention would benefit them. Ireland has already introduced the Irish hip fracture database (IHFD) which has created a benchmark for care. This data created a drive for sustained improvements in clinical standards and cost effectiveness [32]. The IHFD is an established practice in Ireland and has standardised care, with the development of new integrated care teams there is again an opportunity to standardise the care we deliver. The first step needed is to produce a cohesive CGA baseline tool that could be used nationwide and subsequently begin to capture that data in audits thereafter.

This study provides a framework for what is commonly included in CGA documents completed in the community setting in Ireland. These documents should at minimum capture information on physical, psychological, functional, and social–environmental well-being. We believe that including a patient centred question is necessary as well as including a care summary section with a defined action plan*.* The results of this study should inform the generation of a minimum common data set for inclusion in a standardised national CGA baseline tool which could be tailored to the local setting.

Engaging with older persons as key stakeholders to ensure that CGAs are meeting their needs should be prioritised. Co-designing CGAs with patients and carers going forward may be an opportunity to engage with newer technologies and optimise the focus of each patient encounter. Person-centredness is a key principle of integrated care, we need to endorse the successful embedding of this in our day to day practice. The majority, but not all, of the CGAs reviewed included a question clarifying what was most important to the patient. It is essential that CGAs are purposeful, and patient centred.

The drive for best practice in integrated care is relevant in an Irish context but also internationally with more integrated care sites being developed across Europe [33]. This care model organises services in a comprehensive manner; however, there is ongoing work needed to standardize practice and successfully embed person centred care.

## Supplementary Information

Below is the link to the electronic supplementary material.

Supplementary file1 (XLSX 20 KB)
